# Heme Oxygenase-1 Contributes to Both the Engulfment and the Anti-Inflammatory Program of Macrophages during Efferocytosis

**DOI:** 10.3390/cells10030652

**Published:** 2021-03-15

**Authors:** Éva Fige, Judit Szendrei, László Sós, Izabela Kraszewska, László Potor, József Balla, Zsuzsa Szondy

**Affiliations:** 1 Section of Dental Biochemistry, Department of Biochemistry and Molecular Biology, Faculty of Dentistry, University of Debrecen, 4012 Debrecen, Hungary; fige.eva@med.unideb.hu (É.F.); szendreijudit@gmail.com (J.S.); sos.laszlo@med.unideb.hu (L.S.); 2 Department of Medical Biotechnology, Faculty of Biochemistry, Biophysics and Biotechnology, Jagiellonian University, 30-387 Krakow, Poland; izabela.kraszewska@doctoral.uj.edu.pl; 3 HAS-UD Vascular Biology and Myocardial Pathophysiology Research Group, Hungarian Academy of Sciences, University of Debrecen, 4012 Debrecen, Hungary; placi85@gmail.com (L.P.); balla@belklinika.com (J.B.); 4 Department of Pediatrics, Faculty of Medicine, University of Debrecen, 4012 Debrecen, Hungary; 5 Division of Nephrology, Department of Medicine, Faculty of Medicine, University of Debrecen, 4012 Debrecen, Hungary

**Keywords:** heme oxygenase-1, efferocytosis, adenosine A2A receptor, BACH1, inflammation

## Abstract

Heme oxygenase-1 (HO-1) plays a vital role in the catabolism of heme and yields equimolar amounts of biliverdin, carbon monoxide, and free iron. We report that macrophages engulfing either the low amount of heme-containing apoptotic thymocytes or the high amount of heme-containing eryptotic red blood cells (eRBCs) strongly upregulate HO-1. The induction by apoptotic thymocytes is dependent on soluble signals, which do not include adenylate cyclase activators but induce the p38 mitogen-activated protein (MAP) kinase pathway, while in the case of eRBCs, it is cell uptake-dependent. Both pathways might involve the regulation of BTB and CNC homology 1 (BACH1), which is the repressor transcription regulator factor of the HO-1 gene. Long-term continuous efferocytosis of apoptotic thymocytes is not affected by the loss of HO-1, but that of eRBCs is inhibited. This latter is related to an internal signaling pathway that prevents the efferocytosis-induced increase in Rac1 activity. While the uptake of apoptotic cells suppressed the basal pro-inflammatory cytokine production in wild-type macrophages, in the absence of HO-1, engulfing macrophages produced enhanced amounts of pro-inflammatory cytokines. Our data demonstrate that HO-1 is required for both the engulfment and the anti-inflammatory response parts of the efferocytosis program.

## 1. Introduction

The clearance of apoptotic cells by macrophages (efferocytosis) plays a crucial role in maintaining tissue homeostasis and in initiating the resolution of inflammation and tissue repair in case of infection or tissue injury. The process involves five steps: (1) release of ‘find me’ signals by apoptotic cells; (2) recognition of apoptotic cells by macrophages via detecting the summary of apoptotic cell surface changes, especially the appearance of phosphatidylserine; (3) engulfment of apoptotic cells via activating macrophage phagocytic receptors; (4) digestion of the corps; and (5) anti-inflammatory and tissue regeneration promoting the response of macrophages induced by apoptotic cells [1].

Up to now, several ‘find-me’ signals were reported to be released by apoptotic cells in a caspase-dependent manner including lysophosphatidylcholine [2], sphingosine-1-phosphate [3], fractalkine/CX3CL1 [4], and adenosine triphosphate (ATP) [5]. Similarly, numerous macrophage phagocytic receptors expressed in a macrophage-type specific manner were recognized to participate in the recognition and uptake of apoptotic cells, such as integrin β3 and 5, T-cell immunoglobulin and mucin domain containing 4 (Tim-4), transglutaminase 2 (TG2), Mer tyrosine kinase, brain-specific angiogenesis inhibitor 1 (Bai 1), stabilin-2, scavenger receptor A, CD91, CD14, CD36, etc. [6]. The number of ways apoptotic cells induce anti-inflammatory and tissue regeneration promoting response in macrophages is also huge [7,8]. Among these, it has been shown that ATP that is released from apoptotic thymocytes in a caspase-dependent manner [5] is converted to adenosine [9] on the surface of macrophages and triggers cell surface adenosine A_2A_ receptors to promote the anti-inflammatory response [10,11]. In addition, the uptake of apoptotic cells prepares the phagocytosing macrophages to digest the engulfed meal. They do so by upregulating uncoupling protein 2 to release the extra chemical energy generated from the degradation of the apoptotic cell’s material in the form of heat [12], ATP-binding cassette transporter ABCA1 to get rid of the cholesterol taken up [13], and various solute carrier proteins to transport various molecules and metabolites in and out [14], as well as by activating the lipid sensing nuclear receptors, such as the liver X receptor (LXR) and the peroxisome proliferator-activated receptors (PPARs) to alter lipid metabolism [15,16]. 

Recently, by using a global mRNA screen, we have noticed that macrophages engulfing apoptotic thymocytes strongly upregulate heme oxygenase 1, which is an inducible microsomal enzyme that degrades heme to biliverdin, iron, and carbon monoxide (CO) (data not shown). It has been reported that HO-1 not only degrades heme but plays various anti-inflammatory roles in macrophages that are partly dependent, partly independent of the enzymatic activity of the protein [17]. The enzymatic activity-dependent anti-inflammatory effects of HO-1 seem to be mediated by CO [18,19], but the antioxidant properties of biliverdin and the sequestration of ferrous iron by ferritin all concertedly contribute [20]. However, HO-1 can also be localized in the nucleus, where it does not exhibit enzymatic activity, but it modulates the subcellular localization and activity of the transcription factor NF-E2-related factor 2 (Nrf2), impacting metabolic and anti-oxidant defenses [21]. Although several stress activated transcription factor families, such as heat-shock factor (HSF), nuclear factor-κB (NF-κB), nuclear factor–erythroid 2 (NF-E2), and activator protein-1 (AP-1) were shown to participate in the regulation of HO-1 transcription [22], in macrophages, the two main transcription factors that regulates HO-1 transcription are Nrf2 [23] and the signal transducer and activator of transcription 3 (Stat-3), which is activated via interleukin (IL)-10 [24]. 

With no stress stimuli, Nrf2 is accumulated in the cytosol and bound to Kelch-like ECH-associated protein-1 (Keap1). The protein partner inhibits Nrf2 translocation to the nucleus and directs it to proteosomal degradation. However, several cysteine residues of Keap1 are target of oxidative modification resulting in its conformation change and in a consequent release and nuclear translocation of Nrf2 [25]. The activation of a number of MAPK pathways can also promote the nuclear translocation of Nrf2 but without phosphorylating directly the transcription factor [26]. In the nucleus, Nrf2 binds to the small Maf protein, and they together induce the expression of HO-1 via binding to the antioxidant response element (ARE) [27]. On the other hand, HO-1 transcription is negatively regulated by the BACH1 transcription factor, which also interacts with the Maf protein, but they target the Maf recognition element (MARE) to suppress the transcription of HO-1. Heme can release this suppression by targeting BACH1 and inhibiting its binding to MARE [27]. In addition, tyrosine 486 phosphorylation of BACH1 can also promote the nuclear export of BACH1, thus allowing Nrf2 access to the ARE [28]. 

In the present paper, we investigated the mechanism of HO-1 induction and the consequence of the loss of HO-1 during efferocytosis using two different types of dying cells: the low amount of heme-containing thymocytes and the high amount of heme-containing red blood cells (RBCs). 

## 2. Materials and Methods

### 2.1. Reagents 

All reagents were obtained from Sigma-Aldrich (Budapest, Hungary) except when indicated otherwise.

### 2.2. Animals

Most of the experiments were carried out with bone marrow-derived macrophages (BMDMs) differentiated from the bone marrow of 2–5-month-old C57BL/6 mice. In some experiments, BMDMs were differentiated also from BACH1 [29], HO-1 [30], or adenosine A_2A_ receptor (A2AR) [31] or adenosine A_3_ receptor (A3R) [32] knock out mice and from their C57BL/6, C57BL/6xFVB, FVB, and C57BL/6 littermates, respectively. Mice were maintained under specific pathogen-free conditions in the Central Animal Facility, University of Debrecen, except for the HO-1 knock out mice initially provided by Dr. Anupam Agarwal, University of Alabama, Birmingham, USA which were bred and maintained under SPF conditions at the animal facility of the Faculty of Biochemistry, Biophysics and Biotechnology of the Jagiellonian University since 2004. All animal experiments were approved by the Animal Care and Use Committee of the University of Debrecen (DEMÁB) with a permission number 7/2016/DEMÁB.

### 2.3. Generation of Bone Marrow-Derived Macrophages (BMDM)

Mice were sacrificed by isoflurane overdose. Bone marrow progenitors were obtained from the femur of 2 to 5-month-old mice by lavage with sterile physiological saline. Cells were differentiated for 5 days in Dulbecco’s Modified Eagle’s Medium (DMEM) supplemented with 10% fetal bovine serum (FBS), 20% conditioned medium derived from L929 cells [33], as a source for macrophage colony-stimulating factor (M-CSF), 2 mM glutamine, 100 U/mL penicillin, and 100 μg/mL streptomycin at 37 °C in 5% CO_2_. Non-adherent cells were washed away on the third day, and the same culture medium described above was readded. In case of HO-1^+/+^ and ^−/−^ mice for the eryptotic red blood cells (eRBC) phagocytosis and cytokine release experiments, bone marrow progenitors were differentiated for 5 days in DMEM high-glucose medium supplemented with 10% FBS, 10 ng/mL purified human recombinant M-CSF (R&D Systems, Minneapolis, MN, USA), 100 U/mL penicillin, and 100 μg/mL streptomycin at 37 °C in 5% CO_2_.

### 2.4. Generation of Apoptotic Thymocytes and Eryptotic Red Blood Cells (eRBCs)

Thymi were collected from 4-weeks-old C57BL/6 mice, thymocytes were isolated and cultured for 24 h (10^7^ cells/mL) in DMEM supplemented with 2 mM glutamine, 100 U/mL penicillin, and 100 μg/mL streptomycin in the absence of serum to generate apoptotic thymocytes [10]. 

Blood was obtained from C57BL/6 mice under isoflurane anesthesia by supra-orbital punctuation or drained from heart and anticoagulated with heparin. Anticoagulated blood was centrifuged at 211 RCF for 10 min at 4 °C and the plasma and buffy-coat were discarded. RBCs were washed three times with Hanks’ balanced salt solution (5 mM KCl, 0.5 mM KH_2_PO_4_, 135 mM NaCl, 4 mM NaHCO_3_, 0.3 mM Na_2_HPO_4_·2H_2_O, 5 mM glucose, 10 mM HEPES and 2.5 mM CaCl_2_, pH 7.5) at 4 °C and exposed to 1 μg/mL ionomycin for 2.5 h [34]. Around 80% of the resulted cells were Annexin V positive in both cases. The number of cells was determined for each cell type by Bürker chamber cell counting.

### 2.5. Generation of Apoptotic Thymocyte Supernatant 

After 24 h, the cell culture medium, in which apoptotic thymocytes were generated alone or in the presence of 20 μΜ Z-VAD-FMK, was collected after harvesting thymocytes by centrifugation at 211 RCF for 10 min and was filtrated through a 0.22 μm sterile syringe filter (VWR International, cat. number 514-0073, Mississauga, ON, Canada) to remove apoptotic bodies. This supernatant was added to macrophages in DMEM supplemented with 20% FBS in 1:1 ratio to correct for the amount of FBS. 

### 2.6. Efferocytosis Assays

Apoptotic thymocytes were stained with 2.5 µM DeepRed dye (Invitrogen, Carlsbad, CA, USA) for 24 h, while eRBCs were stained with 4 μM of PKH26 Red Fluorescent Cell Linker Kit after the ionomycin treatment according to the protocol provided by the manufacturer. Apoptotic thymocytes and eRBCs were added to 2 × 10^5^ macrophages kept in 2 mL DMEM medium (supplemented with 10% FBS, 2 mM glutamine, 100 U/mL penicillin, and 100 μg/mL streptomycin at 37 °C in 5% CO_2_) on 12-well TPP cell culture plates (Cat. number 92012) in 1:5 macrophage:target cell ratio in the continuous presence or absence of 20 µM SnPPIX (Porphyrin Products, Logan, UT, USA). For short-term phagocytosis assays, after coculture for 1 h, apoptotic cells were washed away. For mid- or long-term phagocytosis assays, macrophages were first exposed to unstained apoptotic cells for 6 h or 24 h, respectively, and then to the stained apoptotic cells for an additional hour. Long-term phagocytosis experiments with eRBCs were performed also by using macrophages generated from HO-1 null mice and their wild-type littermates. Then, macrophages were detached by trypsinization, and their fluorescence was analyzed using FACSCalibur. Macrophages were gated according to their forward and side scatter properties. Engulfing macrophages were identified within the macrophage population based on their high fluorescent emission detected in the FL2 and FL4 channels in the case of PKH26 and DeepRed dye, respectively. For detecting HO-1 expressions, macrophages and target cells without labeling were cultured in a 1:5 target ratio for the indicated times as described above. In some experiments, macrophages were exposed to RpcAMP (100 μM), forskolin (10 μM), or SB203580 (10 μM) 30 min prior to the start of efferocytosis.

### 2.7. Fluorescent Microscopy 

Apoptotic thymocytes were stained with 2.5 µM DeepRed dye (Invitrogen, Carlsbad, CA, USA) for 24 h, while eRBCs were labeled with 4 μM of a PKH26 Red Fluorescent Cell Linker Kit according to the protocol provided by the manufacturer. Apoptotic thymocytes and eryptotic RBCs were added to 2 × 10^5^ C57BL/6 macrophages in a 1:5 macrophage/target cell ratio for 1 h; then, the remaining cells were washed away. Following phagocytosis, BMDMs were fixed by 1% paraformaldehyde and stained with NucBlue Live Cell Stain Ready Probes reagent (Thermo Fisher Scientific, Waltham, MA, USA) according to the manufacturer’s instructions. Pictures were taken on fluorescent microscope (FLoid™ Cell Imaging Station, ThermoFisher, Waltham, MA, USA).

### 2.8. Determination of the Heme Oxygenase Activity in Engulfing and Inhibitor Treated Macrophages

Eryptotic RBCs were added to 10^5^ C57BL/6 macrophages in a 1:5 macrophage/target cell ratio in the presence and absence of 20 µM SnPPIX for 6 h, as it was described in the efferocytosis assays. Then, macrophages were collected from 6 wells, and 10^6^ macrophages were taken up in 800 μL ice-cold PBS and frozen immediately to −70 °C. Heme oxygenase activity was determined later as described by Balla et al. [35].

### 2.9. Quantitative Real-Time Polymerase Chain Reaction (qRT-PCR) Analysis of mRNA Expression 

Total RNA was isolated from BMDMs cultured alone or exposed to apoptotic thymocytes, eRBCs, apoptotic cell supernatants, and various compounds using the TRI reagent according to the manufacturer’s guidelines (ThermoFisher, Waltham, MA, USA). Total RNA was reverse transcribed into cDNA using a High-Capacity cDNA Reverse Transcription Kit (Life Technologies, Budapest, Hungary) according to the manufacturer’s instruction. qRT-PCR was carried out in triplicate using pre-designed FAM-labeled MGB assays (Life Technologies, Budapest, Hungary), including LightCycler 480 Multiwell 384 white plates sealed with adhesive tapes on a Roche LightCycler LC 480 real-time PCR instrument. Relative mRNA levels were calculated using the comparative CT method and were normalized to cyclophilin A mRNA. Catalogue number of the TaqMan assays used for heme oxygenase-1, interleukin-1β, KC, M-CSF, and cyclophilin A were Mm00516005_m1, Mm00434228_m1, Mm04207460_m1, Mm00432686_m1, and Mm02342429_g1, respectively.

### 2.10. Western Blot Analysis

To receive the total cellular proteins, cells were harvested and lysed in cold lysis buffer (see above) The protein content of the samples were determined by Bio-Rad Protein Assay Dye (Bio-Rad, Budapest, Hungary), and then, the supernatant was boiled in loading buffer with an aliquot corresponding to 50–100 μg of protein. Apoptotic thymocyte, eRBC, and BMDM lysates were run on SDS polyacrylamide gels, and the separated proteins were electroblotted onto polyvinylidene difluoride membranes. Membranes were probed with monoclonal anti-HO-1 (MA1-112, ThermoFisher, Waltham, MA, USA) in 1:500, and monoclonal anti-β-actin antibodies (A5441) in 1:10,000 dilution.

### 2.11. Determination of Rac1, Cdc42, and RhoA Activity in Macrophages

Apoptotic thymocytes or eryptotic RBCs were added to 2 × 10^5^ C57BL/6 macrophages in a 1:5 macrophage/target cell ratio in the presence and absence of 20 µM SnPPIX for 24 h as it was described in the efferocytosis assays. Macrophages from 8 wells cultured under the same conditions were collected as one sample, and 1.4 × 10^6^ from them were used for the determinations. Then, the activity of the three G proteins were determined by the G-LISA Rac1, Cdc42, and RhoA activation assay kits (Cytoskeleton Inc., Denver, CO, USA) according to the manufacturer’s instructions.

### 2.12. Determination of Cytokine Production

Wild-type and HO-1 null bone marrow-derived macrophages were plated onto 12-well TPP cell culture plates at a density of 5 × 10^5^ cells/well. To determine cytokine production by macrophages exposed to dying cells, macrophages were exposed to apoptotic thymocytes (isolated from C57BL/6 mice) or eRBCs (isolated from C57BL/6xFVB mice) for 6 h in a 1:5 macrophage/target cell ratio. Then, dying cells were washed away, and the macrophages were cultured for an additional 18 h in DMEM medium. At the end of culture, cell culture media were collected and analyzed by Mouse Cytokine Array (Proteome Profile Array from R&D Systems, Minneapolis, MI, USA). The pixel density in each spot of the array was determined by Image J software.

### 2.13. Statistical Analysis

All the data are representative of at least three independent experiments carried out with macrophages isolated from three different mice. Values are expressed as mean ± S.D. For differences between 2 groups, a two-tailed unpaired Student’s *t*-test was used; for comparisons *n* > 2 groups, a one-way ANOVA (with Tukey’s multiple comparisons test) was used. All statistical analyses were performed using GraphPad Prism 6.01 and a *p* value < 0.05 was considered as significant and is indicated by asterisk (*). 

## 3. Results and Discussion

### 3.1. Both Apoptotic Thymocytes and the High Amount of Heme-Containing Eryptotic Red Blood Cells Induce the Expression of HO-1 in Engulfing Macrophages

**Figure 1:**
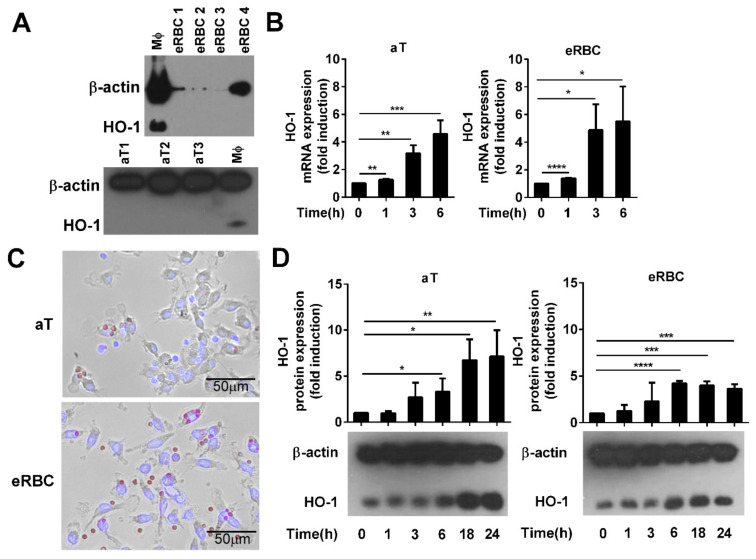
Phagocytosis of apoptotic cells induces the expression of heme oxygenase-1 (HO-1) in engulfing macrophages. (**A**) Lack of detectable HO-1 expression in apoptotic thymocytes (aT) and eryptotic red blood cells (eRBCs) determined by Western blot analysis. β-actin was used as a loading control. MΦ, macrophage. (**B**) Representative fluorescent microscopic images of macrophages engulfing apoptotic thymocytes or eryptotic RBCs. Scale 50 µm. (**C**) Induction of HO-1 expression at mRNA levels in engulfing macrophages exposed to either apoptotic thymocytes or to eryptotic RBCs for the indicated time periods. mRNA expressions were determined by qRT-PCR using cyclophilin as a normalizing gene. Data are fold expressions as compared to the basal HO-1 mRNA expressions in non-engulfing macrophages. (**D**) Induction of HO-1 protein levels in engulfing macrophages exposed to apoptotic thymocytes or eryptotic RBCs for the indicated time periods. Protein levels were determined by Western blot analysis using β-actin as a loading control. One representative Western blot is shown. Data are fold expressions as compared to the basal HO-1 protein expressions in non-engulfing macrophages. Data represent mean ± S.D. (*n* = 3) * *p* < 0.05, ** *p* < 0.01, *** *p* < 0.001, **** *p* < 0.0001.

To investigate the mechanism of HO-1 induction by apoptotic cells in engulfing macrophages and the role of HO-1 in the clearance of dying cells, we selected two types of dying cells: apoptotic thymocytes the heme content, which is below the detection limit [36], and eryptotic red blood cells that contain a very high amount of heme, as hemoglobin makes up about 96% of the red blood cells’ dry content (by weight) [37]. These cells were induced to die as we described previously [10,34]. HO-1 has a strong tissue specific expression [38]. Thus, prior to the experiments, we decided to determine whether apoptotic thymocytes or eRBCs express the HO-1 protein. As seen in Figure 1A, HO-1 protein is not expressed by these cells in such an amount that would interfere with the assays, so they are suitable to study the effect of apoptotic cell uptake on the expression of HO-1 specifically in the engulfing macrophages. 

As seen in Figure 1B,C, independently of their heme content, both types of dying cells induced the mRNA expression of HO-1 in engulfing macrophages within 6 h, and the level of the protein remained elevated even 24 h later. Surprisingly, we have not found a significant difference in the degree of induction during the first 6 h uptake of the two cell types despite the big difference in their heme content.

### 3.2. HO-1 Expression in Engulfing Macrophages Is Induced by Apoptotic Thymocytes via Soluble Signals, While the Induction by Dead RBCs Is Cell Uptake-Dependent

**Figure 2:**
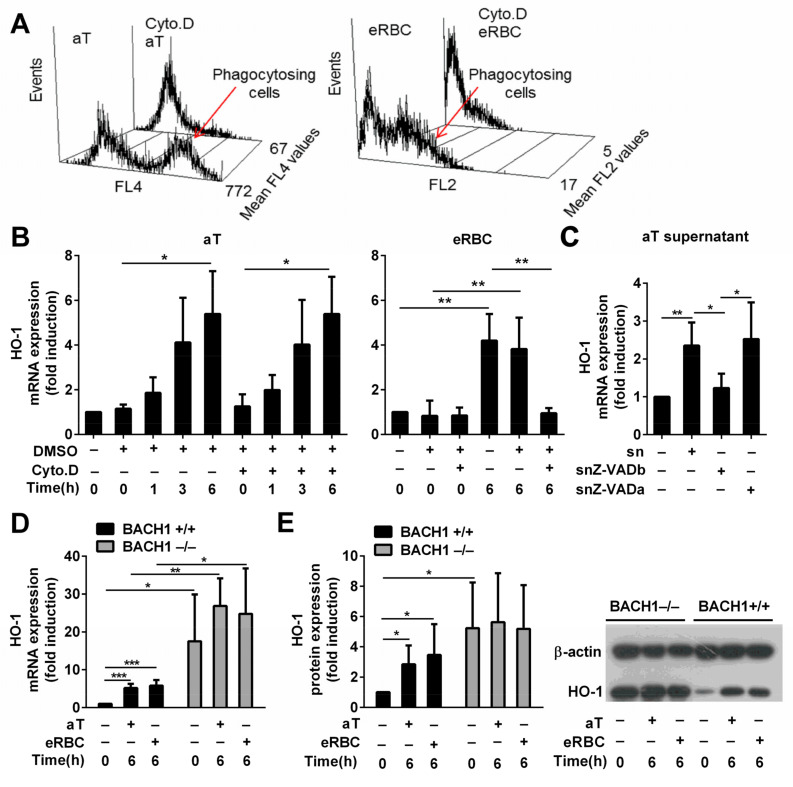
HO-1 expression is BTB and CNC homology 1 (BACH1)-dependent and is induced by soluble signals in macrophages engulfing apoptotic thymocytes, while it is cell uptake-dependent in macrophages engulfing eryptotic RBCs. (**A**) Cytochalasin D, an actin polymerization inhibitor, administered at 5 μM concentration 30 min before phagocytosis inhibits the uptake of both apoptotic thymocytes and eryptotic RBCs determined after 1 h phagocytosis. Data show mean fluorescence intensity within the macrophage population. (**B**) Induction of HO-1 mRNA expression in macrophages engulfing apoptotic thymocytes or eryptotic RBCs in the presence and absence of 5 μM cytochalasin D determined by qRT-PCR using cyclophilin A as a normalizing gene. (**C**) Induction of HO-1 mRNA expression in macrophages exposed to the supernatant of apoptotic thymocytes collected after 24 h serum withdrawal in the presence (snZ-VADb) and absence (sn) of 20 μM Z-VAD-FMK. For determining the Z-VAD-FMK target cell, Z-VAD-FMK was also added directly to the macrophages together with the supernatant generated in the absence of Z-VAD-FMK (snZ-VADa). mRNA expressions were determined at 6 h after supernatant addition by qRT-PCR using cyclophilin A as a normalizing gene. (**D**) Induction of HO-1 mRNA expression in BACH1^+/+^ and BACH1^−/−^ macrophages engulfing apoptotic thymocytes or eRBCs. mRNA expressions were determined by qRT-PCR using cyclophilin A as a normalizing gene. Data are fold expressions as compared to the basal HO-1 mRNA expressions in BACH1^+/+^ macrophages. (**E**) Induction of HO-1 protein expression in BACH1^+/+^ and BACH1^−/−^ macrophages engulfing apoptotic thymocytes or eryptotic RBCs. Protein levels were determined by Western blot analysis using β-actin as a loading control. One representative Western blot is shown. Data are fold expressions as compared to the basal HO-1 protein expressions in BACH1^+/+^ macrophages. Data represent mean ± S.D. (*n* = 3) * *p* < 0.05, ** *p* < 0.01, *** *p* < 0.001.

If the heme content of dead cells plays a role in the induction of HO-1 in engulfing macrophages, the dead cells have to be taken up first. Thus, to assess the involvement of heme in the induction of HO-1 by the apoptotic cell uptake, we decided to block the uptake of apoptotic cells by administering the actin polymerization inhibitor cytochalasin D. As shown in Figure 2A, cytochalasin D in the administered concentration strongly blocked the efferocytosis of both apoptotic thymocytes and RBCs. However, only the eRBC uptake-related HO-1 induction was inhibited in the presence of cytochalasin D; apoptotic thymocytes could still fully induce the expression of HO-1 (Figure 2B).

Since these data indicated that intracellular heme is not the main inducer of HO-1 expression by apoptotic thymocytes, we decided to test whether apoptotic recognition signals are involved. Cytochalasin D inhibits the uptake but does not interfere with the cell surface recognition of dead cells. If we assume that the cell surface signals provided by apoptotic thymocytes and eRBCs are similar (for example, we have reported that they both express the universal cell death signal phosphatidylserine [10,34] that is recognized directly or indirectly by most of the phagocytic receptors in macrophages [39]), these data indicate that eRBCs upregulate HO-1 entirely internally, while apoptotic thymocytes might use only soluble signals. Indeed, the culture medium collected from dying thymocytes was able to induce alone the expression of HO-1 in BMDMs (Figure 2C), while that of eRBCs was not able to do so (data not shown). Although the induction of HO-1 by the apoptotic supernatant was less than that by the apoptotic thymocytes, which could indicate the involvement of a phagocytic receptor that is not triggered by eRBCs, the degradation or dilution of the soluble factor (the supernatant was diluted 1:1 in the macrophage culture), which in the context of efferocytosis is directly released at high concentration around the macrophage, might also explain the observed difference. However, when the supernatant was collected from serum-starved thymocytes incubated in the presence of 20 μM Z-VAD-FMK, a pan-caspase inhibitor, the supernatant was not able to induce HO-1 mRNA expression. In contrast, Z-VAD-FMK added together with the apoptotic thymocyte supernatant did not affect the apoptotic thymocyte supernatant-induced induction of HO-1 mRNA expression, proving that Z-VAD-FMK does not affect the macrophage (Figure 2C). These observations indicate that the soluble signals responsible for HO-1 induction are generated in a caspase-dependent manner in apoptotic thymocytes. Our finding is in harmony with a previous report which indicated that one of the ‘find me’ signals released by apoptotic thymocytes in a caspase-dependent manner, sphingosine-1-phosphate [3], not only regulates migration of macrophages, but is also involved in the upregulation of their HO-1 expression in a p38 MAPK-dependent manner [40]. 

### 3.3. BACH1 Might Be Involved in the Upregulation of HO-1 by Both Apoptotic Thymocytes and eRBCs

If only heme is involved in the upregulation of HO-1 following eryptotic red blood cell uptake in macrophages, this regulation should be completely prevented in the absence of BACH1, since heme was reported to regulate HO-1 expression via targeting the BACH1 transcriptional repressor [27]. It seems that BACH1 has a very strong suppressive effect on the basal expression of HO-1 in BMDMs, since we detected already in the non-treated BACH1 null macrophages a significantly elevated HO-1 expression at both mRNA (Figure 2D) and protein (Figure 2E) levels, as compared to their wild-type controls. As we expected, exposure to eRBCs could not induce further this elevated HO-1 expression (Figure 2D,E). Surprisingly, there was a similar finding when the uptake of apoptotic thymocytes was tested. These observations indicate that either the competition between the two transcription factors, Nrf2 and BACH1, controlling the expression of HO-1 is such an important element in its regulation that when the negative regulator is missing, its transcription is already maximally activated, or both signaling pathways induced by either the dying RBCs or by the dying thymocytes target BACH1. Chromatin immunoprecipitation sequencing experiments detecting potential alterations in the binding of BACH1 to MARE can decide between the two alternatives in the future. Altogether, these data indicate that eRBCs induce HO-1 in engulfing macrophages following their uptake via their heme content, while apoptotic thymocytes use soluble signals, and both signaling pathways might target BACH1.

### 3.4. Adenosine Released during Efferocytosis Is Not Involved in the Induction of HO-1 Expression in Macrophages Engulfing Apoptotic Cells 

During efferocytosis, the ATP released from apoptotic cells in a caspase-dependent manner is converted to adenosine on the surface of macrophages by 5′-ectonucleotidase to trigger adenosine receptors [5,9,10,11]. There are four adenosine receptors, all of which are G protein-coupled receptors. Adenosine A_1_ receptors (A1R) are stimulated by 10^−10^–10^−8^ M concentrations of adenosine and mediate decreases in intracellular cyclic AMP (cAMP) levels, adenosine A_2A_ (A2AR) and A_2B_ receptors (A2BR) are stimulated by higher (5 × 10^−7^ M and 1 × 10^−5^ M, respectively) concentrations of adenosine and mediate increases in cAMP levels, while adenosine A_3_ receptors (A3R) are stimulated by 10^−6^ M concentrations of adenosine and mediate adenylate cyclase inhibition [41]. However, the response of adenosine receptors is also determined by their cell surface expression; thus, when one compares ligand potencies to modulate cAMP levels at comparative receptor densities, it is observed that adenosine is nearly equipotent at A1Rs, A2ARs, and A3Rs, but it is some 50 times less potent at A2BRs [42]. As a result, under physiological conditions, adenosine effects are mediated mainly via A1ARs, A2ARs, and A3Rs. Macrophages have been reported to express A2ARs, A2BRs, and A3Rs [41]. During efferocytosis, A2ARs are up [10], while A3Rs are downregulated [43], indicating that the dominant receptors that mediate the effect of adenosine during efferocytosis at the start of phagocytosis are the A3Rs, while later, they are the A2ARs. Accordingly, previous studies from our and other laboratories have indicated that the adenylate cyclase pathway activated by adenosine A_2A_ receptors contributes to the anti-inflammatory program of apoptotic cell uptake [10,11], while the A3Rs are involved in the chemotactic navigation of macrophages [44]. In addition, reports have indicated that the adenylate cyclase pathway might be involved in the regulation of HO-1 expression [45,46].

**Figure 3:**
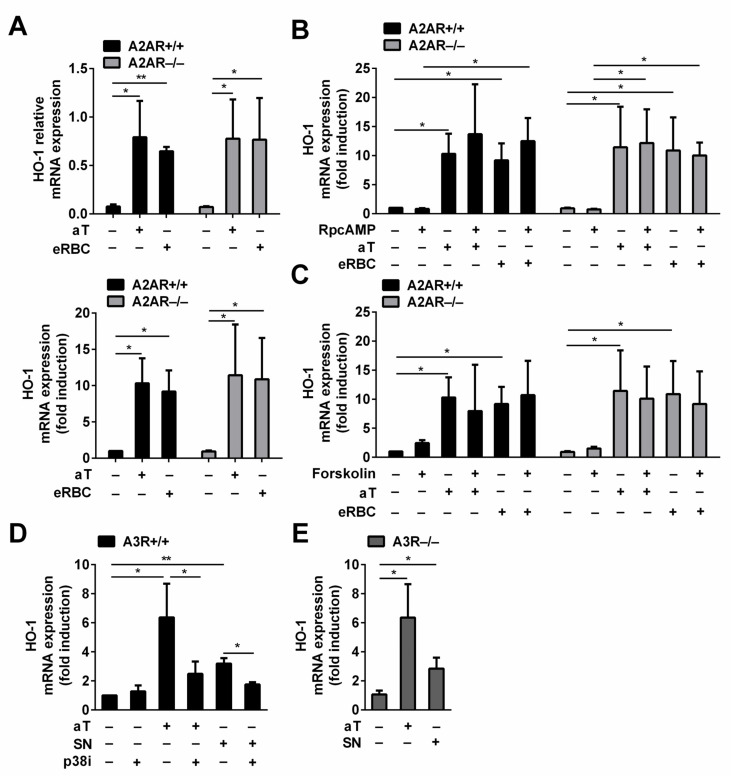
Adenosine is not involved in the induction of HO-1 expression in bone marrow-derived macrophages engulfing apoptotic cells. (**A**) There is no difference in the basal or in the induced HO-1 mRNA expressions in A2AR^−/−^ macrophages as compared to the A2AR^+/+^ macrophages engulfing either apoptotic thymocytes or eryptotic RBCs. Data in the left figure represent relative gene expressions, while those in the right figure represent folds of the non-engulfing A2AR^+/+^ macrophages. (**B**) RpcAMP, a competitive inhibitor of cAMP, added in 100 μM concentration does not affect the basal or the dying cell uptake-induced mRNA expressions of HO-1 in A2AR^+/+^ and A2AR^−/−^ macrophages. Data are expressed as fold of the non-engulfing A2AR^+/+^ macrophages. (**C**) Forskolin, an adenylate cyclase activator, added in 10 μM concentration does not affect either the basal or the dying cell uptake-induced mRNA expressions of HO-1 in A2AR^+/+^ and A2AR^−/−^ macrophages. All cultures contained 0.1 *v*/*v*% DMSO, which is the solvent of the activator. Data are expressed as fold of the non-engulfing A2AR^+/+^ macrophages. (**D**) SB203580, a p38 MAP kinase inhibitor, added in 10 μM concentration, inhibits both the apoptotic thymocyte- and the apoptotic thymocyte supernatant (SN)-induced HO-1 mRNA expressions in A3R^+/+^ macrophages. All cultures contained 0.1 *v*/*v*% DMSO, which is the solvent of the inhibitor. Data are expressed as fold of the non-engulfing A3R^+/+^ macrophages. (**E**) The loss of A3Rs does not influence the apoptotic thymocyte- or the apoptotic thymocyte supernatant-induced HO-1 mRNA expressions detected at 6 h. Data are expressed as fold of the non-engulfing A3R^+/+^ macrophages. mRNA expressions were determined after 6 h phagocytosis or compound exposure by qRT-PCR using cyclophilin A as a normalizing gene. Data represent mean ± S.D. (*n* = 3) * *p* < 0.05, ** *p* < 0.01.

Thus, we decided to investigate whether adenosine also contributes to the soluble signals that regulate HO-1 expression in engulfing macrophages. To answer the question, wild-type and A2AR null macrophages were exposed to either apoptotic thymocytes or dying RBCs, and their HO-1 expressions were compared. As seen in Figure 3A, the loss of A2ARs did not affect the basal HO-1 expression of macrophages. In addition, the loss of A2ARs did not affect the apoptotic thymocyte or the dead RBC-induced HO-1 expression either, indicating that the soluble adenosine signal does not contribute to the apoptotic cell-induced upregulation of HO-1.

Next, we investigated whether the adenylate cyclase pathway contributes to the induction of the HO-1 expression during efferocytosis at all. Thus, we tested the effect of RpcAMP, which is a competitive inhibitor of cAMP [47], on the apoptotic cell-induced HO-1 expression. As seen in Figure 3B, the administration of RpcAMP did not affect either the basal or the induced expression of HO-1 in BMDMs engulfing either dying thymocytes or RBCs independently of their A2AR expression. These data indicate that no other soluble signal is generated during efferocytosis that would contribute to the HO-1 expression via activating the adenylate cyclase pathway. 

Finally, we decided to test whether the adenylate cyclase pathway has any effect on the HO-1 expression in bone marrow-derived macrophages by exposing them to forskolin, which is a strong adenylate cyclase activator [48]. Forskolin added alone or during the phagocytosis of apoptotic cells could not enhance HO-1 expression, indicating that the adenylate cyclase pathway does not contribute to the regulation of HO-1 expression in dead cell engulfing bone marrow-derived macrophages (Figure 3C).

In addition to the inhibition of the adenylate cyclase pathway, A3Rs might activate the p38 MAPK signaling pathway as well in a cell type-dependent manner [49]. Since previous studies indicated the involvement of sphingosine-1-phosphate and also the p38 MAPK pathway by the apoptotic thymocyte supernatant [40], we decided to test whether the apoptotic supernatant-induced HO-1 expression involves the A3Rs. As seen in Figure 3D, inhibition of the p38 MAPK pathway nearly completely prevented the induction of HO-1 in macrophages induced by both the apoptotic thymocyte uptake and by the exposure to the apoptotic thymocyte supernatant. These data confirm the determining role of the p38 MAPK pathway in the apoptotic thymocyte-induced HO-1 expression, and they underline the involvement of soluble signals that trigger the p38 MAPK pathway. However, loss of A3Rs did not affect the induction HO-1 by the apoptotic thymocytes or by their supernatant. Inhibition of the p38 MAP kinase pathway or loss of A3R had no effect on the HO-1 expression induced by the uptake of eRBCs either (data not shown). The different results received following inhibition of the p38 MAP kinase pathway indicate that if both eRBCs and apoptotic thymocytes induce HO-1 mRNA expression in a BACH1-dependent manner, the mechanism of targeting BACH1 is different in the two signaling pathways. Our data altogether exclude the involvement of adenosine in HO-1 induction of engulfing macrophages. 

### 3.5. Loss of HO-1 Activity Results in a Decrease of the Phagocytic Capacity of Macrophages after Long-Term Phagocytosis of Dying Red Blood Cells

**Figure 4:**
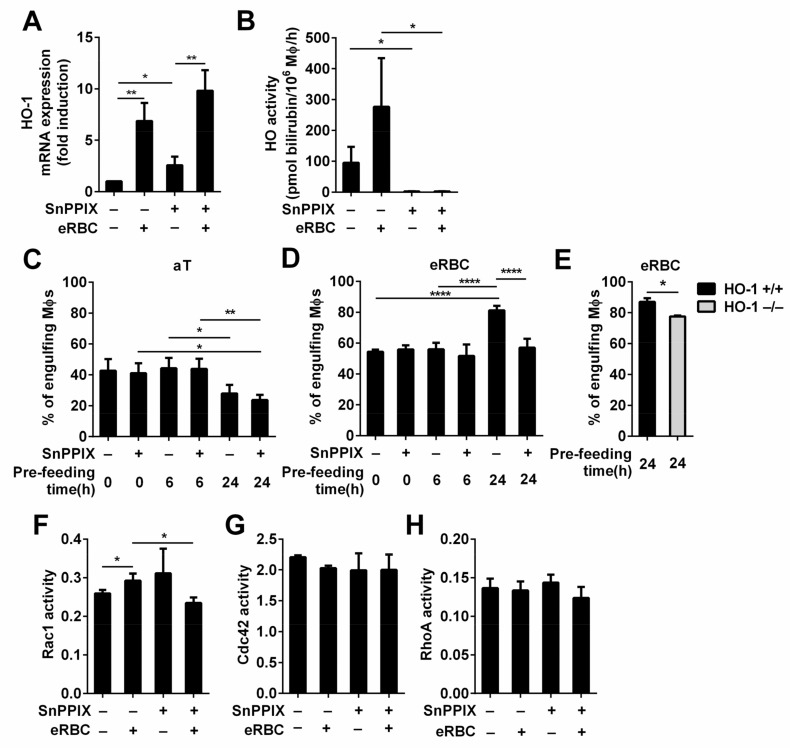
Long-term phagocytic capacity of macrophages engulfing eryptotic red blood cells, but not that of macrophages engulfing apoptotic thymocytes is affected by the loss of HO-1 activity. (**A**) Administration of 20 μM SnPPIX, a competitive inhibitor of HO-1 and HO-2 activities, induces the mRNA expression of HO-1 in macrophages exposed or not to eRBCs detected at 6h by qRT-PCR. Data are expressed as fold of control macrophages. (**B**) 20 μM SnPPIX efficiently blocks HO activities in engulfing and non-engulfing macrophages detected at 6 h following the addition of SnPPIX alone or together with eRBC. (**C**) The apoptotic thymocyte phagocytic capacity of engulfing macrophages was determined after 0, 6, or 24 h of continuous apoptotic thymocyte phagocytosis in the presence or absence of 20 μM SnPPIX. (**D**) eRBC phagocytic capacity of engulfing macrophages was determined after 0, 6, or 24 h continuous eRBC phagocytosis in the presence or absence of 20 μM SnPPIX. (**E**) eRBC phagocytic capacity of engulfing HO-1^+/+^ and HO-1^−/−^ macrophages was determined after 24 h continuous eRBC phagocytosis. (**F**) Activity of Rac1, (**G**) Cdc42, and (**H**) RhoA in macrophages engulfing eryptotic RBCs in the presence and absence of 20 μM SnPPIX for 24 h determined by G-LISA Rac1, Cdc42, and RhoA activation assay kits. In each control experimental set up, 0.26 mM NaOH (pH 7.0), the solvent of SnPPIX, was also added to the culture medium. Data represent mean ± S.D. (*n* = 3 except in D, where *n* = 7) * *p* < 0.05, ** *p* < 0.01, **** *p* < 0.0001.

Next, we investigated whether the loss of HO activity affects the phagocytosis of apoptotic cells. For this purpose, we inhibited HO-1 and HO-2 activities by preincubating macrophages with their competitive inhibitor SnPPIX [50]. Since in line with a previous report [51], SnPPIX induced an increase in the HO-1 mRNA expression in both engulfing and non-engulfing macrophages (Figure 4A), we tested first whether the applied concentration of the inhibitor is sufficient to inhibit the superinduced HO activity in eRBC-exposed macrophages, As seen in Figure 4B, SnPPIX applied in 20 μM concentration efficiently inhibited HO activities. 

The phagocytosis of both apoptotic thymocytes and eryptotic red blood cells was determined immediately after exposure to macrophages (short-term phagocytosis), after continuous phagocytosis for 6 h (mid-term phagocytosis), or after continuous phagocytosis for 24 h (long-term phagocytosis). As it is shown in Figure 4C, the phagocytic capacity of macrophages engulfing apoptotic thymocytes was not affected up to 24 h by SnPPIX, even though both HO activities were inhibited, while the phagocytic capacity of macrophages engulfing the high heme containing RBCs under the same conditions was inhibited at the same time point (Figure 4D). SnPPIX reduced not only the percentage of eRBC engulfing macrophages but also their mean fluorescence from 75.7 +/− 11.9 to 44.8 +/− 8.1 (*p* < 0.01), indicating that the lower number of engulfing macrophages took up less eRBCs. This decrease was not related to a decreased viability of the engulfing macrophages detected by Annexin V-fluorescein isothiocyanate/propidium iodide staining (around 95% in each culture), and it was confirmed by testing the long-term phagocytic capacity of HO-1 null macrophages as well (Figure 4C). These data indicate that the observed decrease was not related to a potential side effect caused by the inhibitor. Our finding is in harmony with a report that has shown that the thymic structure and function of HO-1 null mice is not altered [52], where thymic cells continually die and are cleared, indicating proper heme metabolism, while these mice are characterized by splenomegaly and by a strongly damaged fibrotic structure in the spleen [53], where the majority of aging RBCs are cleared. In these spleens, eRBC-engulfing tissue resident macrophages die due to heme accumulation [53]. The different sensitivity of macrophages to heme accumulation in the two tissues in vivo might be explained by the constant and not altered expression of HO-2 in HO-1^−/−^ macrophages [53,54], the activity of which might be sufficient to handle the low amount of heme generated during efferocytosis of apoptotic thymocytes in the thymus, while not during efferocytosis of the high heme-containing eRBCs in the spleen. Accordingly, when both HOs were inhibited by SnPPIX, we observed a more significant inhibition in the long-term eryptotic red blood cell uptake than in that when HO-1 was lost alone (Figure 4E,F), underlying that the degree of efferocytosis inhibition is dependent on the residual HO activity.

The phagocytosis of apoptotic cells involves numerous phagocytic receptors [6]. Many signals that affect efferocytosis regulate the expression of the phagocytic receptors in macrophages. Thus, we tested whether the engulfment of RBCs in the presence of HO-1 activity inhibitor affects the expression of any of these genes. However, none of the tested key phagocytic receptors or bridging molecules (Tim4, transglutaminase 2, MerTK, integrin β3/β5, stabilin2, CD14, MFG-E8, CD36, thrombospondin) were expressed differently after 24 h of RBC efferocytosis (data are not shown). Thus, the loss of HO activity must inhibit efferocytosis via a different mechanism. 

Signaling pathways initiated by the phagocytic receptors were reported to regulate the GTP loading of several small G proteins to achieve efficient efferocytosis. Thus, the activation of Rac1 and Cdc42 was shown to be required for efferocytosis [55,56], while the activation of RhoA was reported to be inhibitory [57]. Later, using Forster resonance energy transfer biosensors, it was discovered that these small G proteins work in a temporally regulated fashion in which Rac1 and Cdc42 are activated early to work together in facilitating phagocytic cup formation through actin polymerization followed by RhoA activation, which drives mechanical retraction and phagosome internalization [58]. Thus, we decided to determine the general activity of these three G proteins following the 24 h phagocytosis of RBCs in wild-type macrophages exposed or not to SnPPIX. As shown in Figure 4G,H, we found an increase in the amount of activated Rac1 and no alterations in the amount of activated RhoA and Cdc42 after 24 h of RBC efferocytosis. The inhibitor alone did not affect the activity of these G proteins. However, in the presence of the inhibitor, the phagocytosis-induced activation of Rac1 was prevented. Our findings could not confirm the results of a report that indicated that the accumulation of heme in macrophages (in their case during hemolysis) can overactivate Cdc42 by binding to its guanine nucleotide exchange factor dedicated of cytokinesis 8 (DOCK8) [59]. Our data indicate rather that in the absence of HO-1 activity, Rac1 activation is influenced during phagocytosis. Since previous studies demonstrated that carbon monoxide, the product of HO activity, promotes efferocytosis [60], our data might indicate that at physiological levels of heme uptake, the accumulation of heme and loss of production of carbon monoxide together might affect long-term efferocytosis in the absence of HO activity influencing dominantly Rac1 activity.

### 3.6. While Apoptotic Cell Uptake Inhibits Basal Pro-Inflammatory Cytokine Production of Macrophages, Pro-Inflammatory Cytokine Release of Engulfing Macrophages Is Not Altered or Enhanced if HO-1 Protein Is Not Expressed

While the phagocytosis of a variety of pathogenic targets, especially bacteria and virus-infected cells, normally triggers a pro-inflammatory response in macrophages including the production of pro-inflammatory cytokines, the ingestion of apoptotic cells by macrophages usually induces an anti-inflammatory phenotype. Apoptotic cells do not simply fail to provide pro-inflammatory signals; rather, they actively interfere with the inflammatory program [7]. Since it has been reported that HO-1 has anti-inflammatory properties [17,18,19,20], we decided to investigate whether the upregulation of HO-1 contributes to the anti-inflammatory program induced by apoptotic cells by detecting the pro-inflammatory cytokine formation of both dying thymocyte- or RBC-engulfing wild-type and HO-1 null macrophages. Unfortunately, due to the induction of HO-1 (Figure 4A), we could not repeat the experiments in the presence of SnPPIX to investigate separately the effect of the HO-1 activity loss, because we could not have separated the effect of the activity loss and that of the HO-1 protein induction.

**Figure 5:**
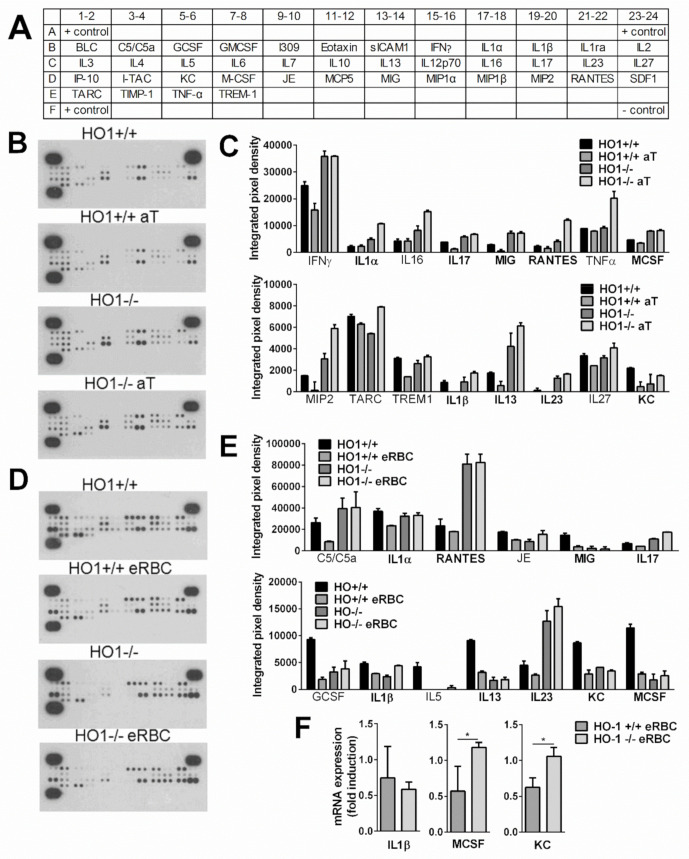
Loss of HO-1 alters the anti-inflammatory effect of apoptotic cell uptake during efferocytosis. (**A**) The map of the 40 cytokines detected on the membranes. (**B**) Cytokine panel of control or apoptotic thymocyte-exposed HO-1^+/+^ and HO-1^−/−^ macrophages. Macrophages were coincubated or not with apoptotic thymocytes for 6 h followed by the removal of apoptotic cells and addition of fresh medium. Supernatants were collected 18 h later, and cytokine levels in the supernatants were analyzed by cytokine array. (**C**) Pro-inflammatory cytokines, the levels of which were suppressed by the apoptotic thymocyte uptake in HO-1^+/+^ but not in HO-1^−/−^ macrophages. (**D**) Cytokine panel of control or eryptotic RBC-exposed HO-1^+/+^ and HO-1^−/−^ macrophages. Macrophages were coincubated or not with eryptotic RBCs for 6 h followed by the removal of apoptotic cells and addition of fresh medium. Supernatants were collected 18 h later, and cytokine levels in the supernatants were analyzed by cytokine array. (**E**) Pro-inflammatory cytokines, the levels of which were suppressed by the eryptotic RBC uptake in HO-1^+/+^ macrophages but not in HO-1^−/−^ macrophages. Bold labels highlight those cytokines that behaved similarly during apoptotic thymocyte and eryptotic RBC uptake. One representative experiment of three is shown. (**F**) Fold changes in the mRNA expressions of IL-1β, MCSF, and KC following 6 h of eRBC uptake in HO-1^+/+^ and ^−/−^ engulfing macrophages detected at 24 following the start of engulfment. mRNA levels were detected by qRT-PCR. Data represent mean ± S.D. (*n* = 3) * *p* < 0.05.

Evaluation of the cytokine secretion profile of unstimulated macrophages was performed using a highly sensitive cytokine antibody array method, enabling the simultaneous detection of low concentrations of multiple cytokines in one assay (picogram per milliliter range). The map of the 40 cytokines detected on the membranes is diagrammed in Figure 5A. The cytokines released were first evaluated by experiments using untreated wild-type and HO-1 null macrophages in vitro. The results reported in Figure 5B–E from non-engulfing macrophages show that most of the available cytokines on the filters were detectable, even though some were at very low levels. The loss of HO-1 did not affect significantly the composition of most of the cytokines released, but we found great individual differences in the amount of pro-inflammatory cytokines produced both within the HO-1^+/+^ and HO-1^−/−^ macrophages derived from different mice. However, as summarized in Figure 5C,E, when macrophages were exposed to either apoptotic thymocytes or eryptotic RBCs, we found several cytokines, the production of which was suppressed in wild-type engulfing macrophages, but increased or remained unaltered in their HO-1 null counterparts. Nine of these cytokines (IL-1α and β, IL-17, monokine induced by gamma (MIG), regulated on activation, normal T cell expressed and secreted (RANTES), MCSF, IL-13, IL-23, keratinocyte chemoattractant, (KC)) highlighted with bold in Figure 5C,E were identified during both thymocyte and RBC phagocytosis as pro-inflammatory cytokines, the downregulation of which was affected by the loss of HO-1 in three independent experiments. Interestingly, we found the IL-1R antagonist to respond in a similar manner, not only in this study, but also in a previous study [10], which is in line with the observation that IL-1 production and activity are regulated separately, and an innate memory might also influence IL-1 action [61]. 

To confirm our results further, we selected three of these cytokines to determine their mRNA levels in the same macrophages from which the supernatants were collected. Since the individual differences in cytokine productions were reflected in individual differences in the mRNA levels as well, we demonstrated their change as fold expression following eRBC uptake. As shown in Figure 5F, the mRNA expressions of MCSF and KC were also decreased in eRBC-exposed wild type but not in HO-1 null macrophages. We did not find the same for IL-1β. However, IL-1β protein levels are also affected by the degree of caspase-1 activation, and HO-1 was shown to attenuate NOD-like receptor containing a pyrin domain 3 (NLRP3) inflammasome activity [62].

Altogether, our data indicate that the induction of HO-1 contributes to the anti-inflammatory effects induced by dying cells in engulfing macrophages.

## 4. Conclusions

Macrophages perform two main roles during efferocytosis of healthy dying cells: (1) engulfment and degradation of the dying cell and (2) suppression of inflammation. In this paper, we have investigated the role of HO-1, which is an enzyme that degrades heme, in macrophages engulfing two dying cell types, the low amount of heme-containing apoptotic thymocytes and the high amount of heme-containing eryptotic RBCs. Altogether, the data presented here indicate that dying cells upregulate HO-1 in macrophages: apoptotic thymocytes via soluble signals that do not include adenosine or other cAMP elevating ones but require the p38 MAPK signaling pathway, while eRBCs following their uptake. Both regulatory pathways might involve the regulation of BACH1. In case of the inhibited HO activity, the uptake of those cells that have low heme content did not affect the phagocytic capacity of macrophages even after 24 h continuous engulfment, while the same resulted in an inhibition of phagocytosis in case of high heme-containing cells’ uptake. In the latter case, the loss of HO activity affected the engulfment-induced Rac1 activation. While apoptotic cell uptake suppressed the basal pro-inflammatory cytokine production of wild-type macrophages, in the absence of HO-1, we detected an enhanced or unaltered pro-inflammatory cytokine release during the engulfment of both cell types. The anti-inflammatory effect of HO-1 might explain why even low heme-containing dying cells upregulate HO-1 in macrophages and why they use alternative signals than intracellular heme accumulation to act so. Our data altogether demonstrate that HO-1 contributes to both the engulfment and the anti-inflammatory program of macrophages during efferocytosis. 

## Data Availability

The data presented in this study are available upon request from the corresponding author.
